# Challenges of Hepatitis B Virus Reactivation and CD19 Testing Following Tafasitamab Plus Lenalidomide for Relapsed Diffuse Large B-Cell Lymphoma

**DOI:** 10.14740/jmc5313

**Published:** 2026-03-27

**Authors:** Ta-Chuan Yu, Wen-Chien Chou

**Affiliations:** a Department of Internal Medicine, National Taiwan University Hospital, Yun-Lin Branch, Douliu City, Yunlin County 640, Taiwan, Republic of China; b Division of Hematology, Department of Internal Medicine, National Taiwan University Hospital, Taipei City 100, Taiwan, Republic of China

**Keywords:** Diffuse large B-cell lymphoma, Hepatitis B virus, Tafasitamab, Lenalidomide

## Abstract

The outcomes of transplant-ineligible patients with relapsed/refractory (R/R) diffuse large B-cell lymphoma (DLBCL) are generally poor but much improved by novel targeted therapies and immunotherapies. Tafasitamab plus lenalidomide is an approved regimen for patients with R/R DLBCL. Nonetheless, the clinical data regarding Asian populations and hepatitis B virus (HBV) carriers are lacking in both the pivotal L-MIND and real-world studies. We present an 81-year-old HBV carrier with relapsed DLBCL, who achieved and continued a complete metabolic response (CMR) during 1.5 years of tafasitamab plus lenalidomide as second-line therapy. Notably, an episode of HBV reactivation occurred after four cycles of tafasitamab plus lenalidomide, which was early detected and successfully managed with preemptive nucleotide analogues. Interestingly, CD19 was not detectable by flow cytometry after 10 cycles of treatment despite continuous control of the disease. CD19 expression may be diminished by exams using a tafasitamab-competitive-binding clone. This case highlights not only the concern of HBV reactivation, but also the diagnostic challenge due to CD19 epitope masking following tafasitamab therapy.

## Introduction

Diffuse large B-cell lymphoma (DLBCL) is the most common subtype of lymphoma, accounting for 30% of all lymphoma cases worldwide [1]. The standard first-line treatment of this aggressive lymphoid malignancy is high-dose immunochemotherapies, namely rituximab, cyclophosphamide, doxorubicin, vincristine, and prednisone (R-CHOP), as well as polatuzumab vedotin, rituximab, cyclophosphamide, doxorubicin, and prednisone (Pola-R-CHP). Nonetheless, only 65% of patients are cured by first-line therapy, highlighting the importance of salvage therapies [1]. The conventional chemotherapy-based salvage therapy consists of high-dose chemotherapies followed by autologous stem cell transplant (ASCT). For those ineligible for ASCT, outcomes remain dismal. In a Canadian real-world study, the median overall survival (OS) in patients with relapsed/refractory (R/R) DLBCL who did not receive ASCT was only 8.6 months [2]. Recently, developments in targeted therapies, bispecific T-cell engagers, and chimeric antigen receptor T cells have brought hope to improve their outcome.

Tafasitamab is an Fc-modified humanized anti-CD19 monoclonal antibody that binds CD19 Fcγ receptor, triggering antibody-dependent cellular cytotoxicity and phagocytosis in malignant B cells [3–5]. Lenalidomide is an immunomodulator that stimulates natural killer/T-cell function and has direct anti-tumor activity. The clinical efficacy of tafasitamab plus lenalidomide in transplant-ineligible adults with R/R DLBCL was evaluated in the phase II L-MIND study (NCT02399085), in which 43% of patients achieved complete responses (CRs) and 18% had partial responses (PRs) [5]. Based on this result, tafasitamab plus lenalidomide was approved for the treatment of ASCT-ineligible patients with R/R DLBCL by the US Food and Drug Administration in 2020. In the 5-year analysis of L-MIND study, treatment responses were maintained, with 5-year progression-free survival (PFS) of 36.1% and OS of 40.3% [6]. The median durations of PFS and OS were 11.6 and 33.5 months, respectively [7]. A real-world study from Spain also supported the anti-tumor activity of tafasitamab plus lenalidomide, with an overall response rate between 51% and 61%, and a CR rate between 35% and 42% [8]. Treatment toxicities of tafasitamab plus lenalidomide were generally considered manageable, with the most common grade 3–4 adverse events being cytopenia and infections [5, 8].

Besides lymphoma control, patients with resolved hepatitis B virus (HBV) infection or HBV carriers receiving B-cell-depleting therapies have a high risk (> 10%) of HBV reactivation [9–14]. In these patients, antiviral prophylaxis is a routine practice while they receive CD20-targeting therapy. As a CD19-targeting B-cell depleting therapy, tafasitamab theoretically carries risks of HBV reactivation. Despite the shared mechanism between CD19- and CD20-targeting therapies, data on HBV reactivation risk with tafasitamab plus lenalidomide are limited. In the pivotal L-MIND study, patients with positive hepatitis B surface antigen (HBsAg) were excluded. The prevalence of chronic HBV infection varies widely, from the lower prevalence (< 0.9%) in Europe and North America to higher prevalence in southeastern Asia and Africa (> 8%) [15]. As a result, there are no data in clinical trials or real-world studies reporting HBV reactivation in tafasitamab plus lenalidomide-treated patients [8, 16].

To mitigate the gap in the under-characterized risk of HBV reactivation with tafasitamab plus lenalidomide, we report an 81-year-old Asian HBV carrier with relapsed DLBCL after 17 months of remission following R-CHOP therapy. After tafasitamab plus lenalidomide salvage, her DLBCL achieved a second complete metabolic remission (CMR), which was maintained for at least 1.5 years. Interestingly, one episode of HBV reactivation developed after four cycles of tafasitamab plus lenalidomide, which was successfully managed with nucleotide analogues. In addition, CD19 expression on marrow clonal B cells became negative after 10 cycles of tafasitamab. Given persistent lymphoma control, tafasitamab-related CD19 epitope masking is considered rather than CD19 antigen loss. This case demonstrates the antitumor activity, the risk of HBV reactivation, and the diagnostic challenges posed by CD19 epitope masking with tafasitamab plus lenalidomide, for which data are lacking in the medical literature.

## Case Report

**Figure 1:**
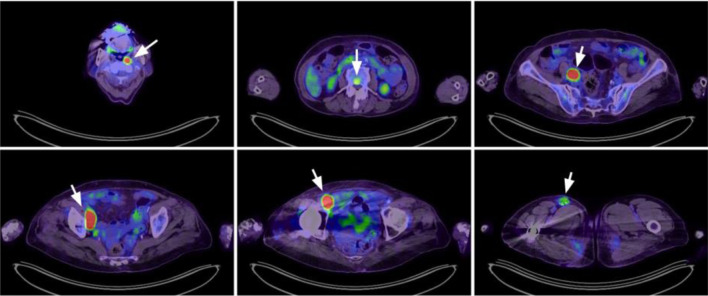
Positron emission tomography scan at diagnosis revealed lymphoma involvement at multiple nodal areas (left nasopharyngeal, right iliac, right femoral) and vertebral spine (arrowheads).

A 79-year-old Asian woman was initially presented with an enlarging right femoral mass for 2 months. An excisional biopsy confirmed the diagnosis of DLBCL, with immunohistochemical expression of MUM-1, BCL-6, BCL-2, and c-MYC. A positron emission tomography (PET) scan revealed stage IV disease with multiple bone involvements (Fig. 1). Bone marrow study revealed 0.1% of clonal B cells that expressed CD19, CD20, and surface kappa light chain. Based on her age, extranodal involvement, and stage IV disease, her revised International Prognostic Index (R-IPI) was 3.

Viral hepatitis profiles were screened at diagnosis, revealing positive for HBsAg and hepatitis B core antibody (anti-HBc), while negative for hepatitis B surface antibody (anti-HBs), hepatitis B e-antibody (anti-HBe), and hepatitis C virus antibody. Induction therapy as R-miniCHOP was initiated, with tenofovir alafenamide as prophylaxis for HBV reactivation. To achieve better disease control, the following five cycles of chemoimmunotherapy were escalated to standard R-CHOP. Interim computed tomography (CT) scan after cycle 3 revealed a CR, and a PET scan after end-of-treatment revealed a CMR. The patient then received regular follow-up at the outpatient clinic. Prophylactic tenofovir alafenamide was discontinued 6 months after end-of-treatment.

### Second remission achieved by tafasitamab plus lenalidomide

**Figure 2:**
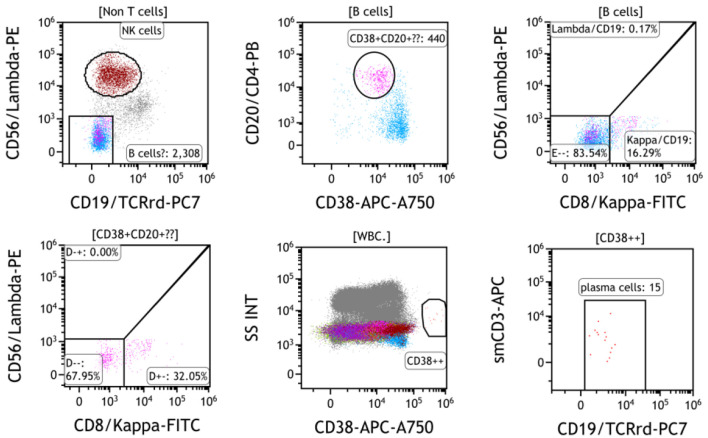
Bone marrow flow cytometry at cycle 10 day 6 of tafasitamab plus lenalidomide showed a small population of CD19-negative B cells (purple, 440 events, 0.16%) expressing CD20 and surface kappa light chain. Plasma cells are nearly depleted (15 events, 0.01% of marrow cells).

Seventeen months after the end-of-treatment, she returned to our hospital for a fever of unknown origin. The results of a serial workup confirmed extranodal DLBCL relapse in the axial, appendicular skeletons, and bone marrow. Considering her frail status, high-dose chemotherapy with ASCT was not chosen. Rituximab plus lenalidomide (R-square) resolved her fever. One month later, the treatment was bridged to tafasitamab plus lenalidomide from a compassionate use program in Taiwan. Lenalidomide dose was adjusted (5 mg every other day, 21 days a month) for grade 4 neutropenia. A PET scan after six cycles of tafasitamab-lenalidomide treatment revealed a CMR. A bone marrow study on the sixth day of cycle 10 tafasitamab plus lenalidomide revealed few (0.16 %) clonal B cells remaining in marrow. Notably, these clonal B cells were still positive for CD20 and surface kappa light chain, but negative for CD19 expression (tested by CD19 clone J3-119), as shown in Figure 2. Plasma cells in her marrow are nearly depleted (0.01%), and hypogammaglobulinemia (IgG < 600 mg/dL) developed after four cycles of tafasitamab plus lenalidomide. The antitumor effect is long-lasting, and this patient remained on treatment 1.5 years after her DLBCL relapse.

### HBV reactivation during tafasitamab plus lenalidomide

In Taiwan, prophylactic nucleos(t)ide analogues are reimbursed when patients receive rituximab, anthracycline-based chemotherapy, or medium-high dose steroids (defined as prednisolone ≥ 20 mg/day for more than 4 weeks). As a chronic HBV carrier, this patient received tenofovir alafenamide as HBV reactivation prophylaxis during first-line R-CHOP treatment but was omitted during tafasitamab-lenalidomide treatment.

**Figure 3:**
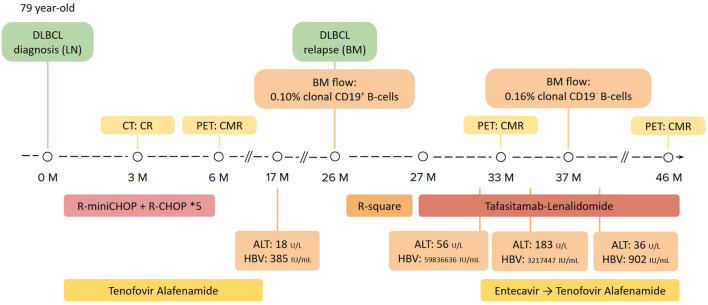
The summary of the patient’s lymphoma and HBV treatment course. Images and laboratory exams of disease diagnosis and staging are shown in the upper part. Lymphoma treatment regimens are shown in the middle, whereas viral hepatitis profiles and HBV treatments are summarized in the lower part. BM: bone marrow; HBV: hepatitis B virus; LN: lymph node; R-square: rituximab plus lenalidomide.

After four cycles of tafasitamab plus lenalidomide without HBV prophylaxis, this patient developed hypogammaglobulinemia, with a serum IgG concentration of 576 mg/dL. Her alanine aminotransferase (ALT) mildly increased to 56 U/L, with serum HBV viral load increased from 32 IU/mL at DLBCL relapse to 59,836,636 IU/mL. History taking and reviewing her medications did not reveal a possible cause of her abnormal ALT level other than HBV infection. Out-of-pocket preemptive entecavir was prescribed for HBV reactivation. One month later, her ALT further increased to 183 U/L, but her serum HBV viral load dropped to 3,217,447 IU/mL. Serum total bilirubin levels were normal during this period, compatible with a diagnosis of HBV reactivation without hepatic decompensation. Under continued HBV treatment, her ALT and HBV viral load then gradually improved. The whole treatment course was summarized in Figure 3.

## Discussion

We reported an 81-year-old HBV carrier with relapsed DLBCL, who achieved CMR after tafasitamab plus lenalidomide. The remission was long-standing, persisting at least 1.5 years. After 10 cycles of tafasitamab plus lenalidomide, CD19 expression was not detected on marrow clonal B cells. Without HBV prophylaxis, one episode of HBV reactivation occurred during tafasitamab plus lenalidomide, but was successfully managed with preemptive HBV treatment.

B-cell-depleting therapies are well-established high-risk factors for HBV reactivation. Without primary prophylaxis, HBV reactivation develops in more than 10% of HBV-infected patients receiving B-cell-depleting therapies [10–15, 17, 18]. There are two main mechanisms of HBV reactivation after B-cell-depleting therapies: impaired humoral immunity and depletion of antigen-presenting B cells [19, 20]. These data are primarily derived from patients with lymphoid malignancies or autoimmune disorders who are receiving anti-CD20 monoclonal antibodies (rituximab, obinutuzumab, ofatumumab). Since CD20 is expressed on mature B cells, the use of anti-CD20 monoclonal antibodies depletes mature antigen-presenting B cells and downstream immunoglobulin-secreting plasma cells. Rituximab induces a marked and sustained depletion of peripheral B cells for 6–9 months, leaving only CD20-negative plasmablasts, which are insufficient for ongoing anti-HBs production [21]. The disrupted immune surveillance of HBV-infected hepatocytes results in the reactivation of HBV, which is inversely correlated with the level of serum anti-HBs [17]. Meanwhile, the depletion of B cells impairs the priming of hepatitis B core antigen-specific, B-cell-dependent cytotoxic T cells [22]. Currently, data on the risks of HBV reactivation with B-cell-depleting monoclonal antibodies other than CD20-directed therapies are limited. Nonetheless, given the similar mechanism of B-cell depletion, HBV prophylaxis is mandatory for HBV-infected patients receiving any B-cell-depleting therapy.

In addition to tafasitamab, lenalidomide also carries the risk of HBV reactivation. Lenalidomide is an immunomodulatory drug that works through cereblon-mediated degradation of transcription factors, leading to both direct tumoricidal effects and immunomodulation by activating natural killer/T cells [23–26]. While the precise mechanisms by which lenalidomide precipitates HBV reactivation remain incompletely characterized, lenalidomide has been associated with 8% risk of HBV reactivation in clinical practice [27].

This is the first case report presenting HBV reactivation in patients treated with tafasitamab plus lenalidomide. Tafasitamab is a monoclonal antibody that targets CD19, an antigen expressed across early B-cell precursors to late-stage plasma cells. The targeting of CD19-expressing cells is supported by findings in our patient, who had hypogammaglobulinemia and depleted plasma cells in marrow after tafasitamab plus lenalidomide treatment. For HBV-infected patients receiving B-cell-depleting agents, both the Asian Pacific Association for the Study of the Liver (APASL) and the European Association for the Study of the Liver (EASL) recommend prophylactic antiviral therapy to prevent HBV reactivation [28, 29]. Nonetheless, in Taiwan, HBV prophylaxis is reimbursed only for patients receiving rituximab. This case indicates the risk of HBV reactivation with B-cell-depleting agents is class-specific, and the requirement for HBV prophylaxis should extend beyond rituximab. Since both tafasitamab and lenalidomide carry the risk of HBV reactivation, HBV prophylaxis should be administered in patients treated by this regimen. In patients who cannot receive HBV prophylaxis, HBV viral load should be regularly monitored to detect HBV reactivation early.

### Competitive binding of tafasitamab challenges the detection of CD19

Loss of CD19 expression after CD19-targeting therapies in patients with DLBCL is a well-recognized mechanism of resistance, particularly following CD19-directed chimeric antigen receptor T-cell (CAR-T) therapy [30–32]. In tafasitamab, loss of CD19 expression after treatment has not been observed. One study group enrolled tumor biopsies from 64 patients receiving tafasitamab in the L-MIND study, trials for indolent lymphomas, and real-world settings [33, 34]. These samples were taken after a median of 24 days following tafasitamab infusion. Immunohistochemistry showed that CD19 expression was maintained, with no evidence of CD19 mutation, exon skipping, or loss of CD19 mRNA expression by DNA and RNA analyses. Another real-world study reported nine patients who received tafasitamab prior to CD19 CAR-T therapy [35]. After a median of 7 days following tafasitamab infusion, CD19 expression was maintained in all tested cases (n = 3). CAR-T responses were observed in seven out of nine patients (four CRs, three PRs). Despite the retention of CD19 after tafasitamab treatment, competitive bindings of CD19 epitope were reported between tafasitamab and various CD19-detecting clones used in immunohistochemical stains (BT51E, LE-CD19, D4V4E) as well as flow cytometry (HIB19, 4G7, FMC63, SJ5C1, J3-119, LT19, REA675, HD37) [36]. The competitive binding of tafasitamab will reduce or diminish the capabilities of these clones to detect CD19 [36, 37]. A case report (n = 2) demonstrated reemergence of CD19 after prior CD19-negative test results [37]. Another study found that CD19 expression after tafasitamab is schedule-dependent, with all biopsies performed more than 3 weeks after the last tafasitamab infusion being CD19-positive [38]. In tafasitamab-treated samples, acidic dissociation of tafasitamab from CD19, or a washout period of more than 3 weeks have been proposed to reduce the risk of false negativity [36, 38]. In our case, clonal B cells involved bone marrow at both DLBCL relapse and after tafasitamab treatment. Using a flow cytometry with CD19 clone J3-119, CD19 was not detected 6 days after 10th cycle of tafasitamab infusion. Nonetheless, the result of the following PET scan remained in CMR, favoring CD19 masking rather than loss. Whether it is true CD19 antigen loss or tafasitamab-related CD19 epitope masking remains to be confirmed.

### Conclusion

In this case report, we present an HBV carrier with relapsed DLBCL who achieved durable remission under tafasitamab plus lenalidomide. This case demonstrated the risk of HBV reactivation of tafasitamab plus lenalidomide treatment without HBV prophylaxis, for which data are lacking in published literature. This case also highlights the diagnostic challenge of CD19 expression after tafasitamab. To avoid misinterpretation of CD19 expression after tafasitamab, further studies are needed to optimize the diagnostic protocol.

## Data Availability

The data supporting the findings of this study are available from the corresponding author upon reasonable request.

## References

[1] Sehn LH, Salles G (2021). Diffuse large B-cell lymphoma. N Engl J Med.

[2] Peters A, Nowakowski GS, Dabas R, Amoloja T, Xue Z, Koch C, Waltl EE (2025). Management of Canadian patients with refractory or relapsed diffuse large B-cell lymphoma in the real world: a subanalysis of the RE-MIND2 study. Oncologist.

[3] Horton HM, Bernett MJ, Pong E, Peipp M, Karki S, Chu SY, Richards JO (2008). Potent in vitro and in vivo activity of an Fc-engineered anti-CD19 monoclonal antibody against lymphoma and leukemia. Cancer Res.

[4] Awan FT, Lapalombella R, Trotta R, Butchar JP, Yu B, Benson DM, Roda JM (2010). CD19 targeting of chronic lymphocytic leukemia with a novel Fc-domain-engineered monoclonal antibody. Blood.

[5] Salles G, Duell J, Gonzalez Barca E, Tournilhac O, Jurczak W, Liberati AM, Nagy Z (2020). Tafasitamab plus lenalidomide in relapsed or refractory diffuse large B-cell lymphoma (L-MIND): a multicentre, prospective, single-arm, phase 2 study. Lancet Oncol.

[6] Duell J, Abrisqueta P, Dreyling MH (2023). Five-year subgroup analysis of tafasitamab + lenalidomide from the phase II L-MIND study in patients with relapsed or refractory diffuse large B-cell lymphoma. J Clin Oncol.

[7] Duell J, Abrisqueta P, Andre M, Gaidano G, Gonzales-Barca E, Jurczak W, Kalakonda N (2024). Tafasitamab for patients with relapsed or refractory diffuse large B-cell lymphoma: final 5-year efficacy and safety findings in the phase II L-MIND study. Haematologica.

[8] Gutierrez A, Zeberio I, Penalver FJ, Martinez-Barranco P, Perez S, Morillo D, Martin X (2025). Tafasitamab plus lenalidomide as salvage therapy in diffuse large B-cell lymphoma: real-world experience from GELTAMO. Blood Adv.

[9] Reddy KR, Beavers KL, Hammond SP, Lim JK, Falck-Ytter YT, American Gastroenterological Association I (2015). American Gastroenterological Association Institute guideline on the prevention and treatment of hepatitis B virus reactivation during immunosuppressive drug therapy. Gastroenterology.

[10] Pei SN, Chen CH, Lee CM, Wang MC, Ma MC, Hu TH, Kuo CY (2010). Reactivation of hepatitis B virus following rituximab-based regimens: a serious complication in both HBsAg-positive and HBsAg-negative patients. Ann Hematol.

[11] Seto WK, Chan TS, Hwang YY, Wong DK, Fung J, Liu KS, Gill H (2014). Hepatitis B reactivation in patients with previous hepatitis B virus exposure undergoing rituximab-containing chemotherapy for lymphoma: a prospective study. J Clin Oncol.

[12] Kusumoto S, Arcaini L, Hong X, Jin J, Kim WS, Kwong YL, Peters MG (2019). Risk of HBV reactivation in patients with B-cell lymphomas receiving obinutuzumab or rituximab immunochemotherapy. Blood.

[13] Tsai YF, Yang CI, Du JS, Lin MH, Tang SH, Wang HC, Cho SF (2019). Rituximab increases the risk of hepatitis B virus reactivation in non-Hodgkin lymphoma patients who are hepatitis B surface antigen-positive or have resolved hepatitis B virus infection in a real-world setting: a retrospective study. PeerJ.

[14] Hou KC, Su TH, Kao CN, Cheng HR, Tseng TC, Liu CJ, Hsieh SC (2024). Rituximab carries high risks of hepatitis B virus reactivation in hematologic and rheumatic patients with chronic or resolved hepatitis B. J Gastroenterol Hepatol.

[15] Jeng WJ, Papatheodoridis GV, Lok ASF (2023). Hepatitis B. Lancet.

[16] You J, Chen W, Yan Z, Tian D, Yi H, Feng Y, Zhang M (2026). Anti-CD19 antibody tafasitamab therapy for relapsed or refractory diffuse large B-cell lymphoma: a case series. Anticancer Drugs.

[17] Guo YF, Pan JX, Zhuang WH (2018). Concurrent and reactivation of hepatitis B virus infection in diffuse large B-cell lymphoma: risk factors and survival outcome. Infect Agent Cancer.

[18] Oh MJ, Lee HJ (2013). A study of hepatitis B virus reactivation associated with rituximab therapy in real-world clinical practice: a single-center experience. Clin Mol Hepatol.

[19] Mak JWY, Law AWH, Law KWT, Ho R, Cheung CKM, Law MF (2023). Prevention and management of hepatitis B virus reactivation in patients with hematological malignancies in the targeted therapy era. World J Gastroenterol.

[20] Vanwolleghem T, Groothuismink ZMA, Kreefft K, Hung M, Novikov N, Boonstra A (2020). Hepatitis B core-specific memory B cell responses associate with clinical parameters in patients with chronic HBV. J Hepatol.

[21] Leandro MJ (2013). B-cell subpopulations in humans and their differential susceptibility to depletion with anti-CD20 monoclonal antibodies. Arthritis Res Ther.

[22] Lazdina U, Alheim M, Nystrom J, Hultgren C, Borisova G, Sominskaya I, Pumpens P Priming of cytotoxic T cell responses to exogenous hepatitis B virus core antigen is B cell dependent. J Gen Virol. 2003;84(Pt.

[23] Fink EC, Ebert BL (2015). The novel mechanism of lenalidomide activity. Blood.

[24] Gribben JG, Fowler N, Morschhauser F (2015). Mechanisms of action of lenalidomide in B-cell non-Hodgkin lymphoma. J Clin Oncol.

[25] Noonan K, Rudraraju L, Ferguson A, Emerling A, Pasetti MF, Huff CA, Borrello I (2012). Lenalidomide-induced immunomodulation in multiple myeloma: impact on vaccines and antitumor responses. Clin Cancer Res.

[26] Semeraro M, Vacchelli E, Eggermont A, Galon J, Zitvogel L, Kroemer G, Galluzzi L (2013). Trial Watch: Lenalidomide-based immunochemotherapy. Oncoimmunology.

[27] Ataca Atilla P, Yalciner M, Atilla E, Idilman R, Beksac M (2019). Hepatitis B reactivation rate and fate among multiple myeloma patients receiving regimens containing lenalidomide and/or bortezomib. Turk J Haematol.

[28] Lau G, Yu ML, Wong G, Thompson A, Ghazinian H, Hou JL, Piratvisuth T (2021). APASL clinical practice guideline on hepatitis B reactivation related to the use of immunosuppressive therapy. Hepatol Int.

[29] European Association for the Study of the Liver (2025). EASL Clinical practice guidelines on the management of hepatitis B virus infection. J Hepatol.

[30] Sworder BJ, Kurtz DM, Alig SK, Frank MJ, Shukla N, Garofalo A, Macaulay CW (2023). Determinants of resistance to engineered T cell therapies targeting CD19 in large B cell lymphomas. Cancer Cell.

[31] Frank MJ, Baird JH, Kramer AM, Srinagesh HK, Patel S, Brown AK, Oak JS (2024). CD22-directed CAR T-cell therapy for large B-cell lymphomas progressing after CD19-directed CAR T-cell therapy: a dose-finding phase 1 study. Lancet.

[32] Lownik J, Boiarsky J, Birhiray R, Merchant A, Mead M (2024). Sequencing of anti-CD19 therapies in the management of diffuse large B-cell lymphoma. Clin Cancer Res.

[33] Duell J, Obr A, Augustin M, Endell J, Liu H, Geiger S, Silverman IM (2022). CD19 expression is maintained in DLBCL patients after treatment with tafasitamab plus lenalidomide in the L-MIND study. Leuk Lymphoma.

[34] Duell J, Alvarez Arias D, Klapper W (2025). CD19 expression is preserved following CD19-directed monoclonal antibody therapy with tafasitamab. Blood.

[35] Epperla N, Nastoupil LJ, Feinberg B, Galvin J, Pathak P, Amoloja T, Gentile D (2025). Real-world use of tafasitamab preceding CD19-directed chimeric antigen receptor T-cell therapy for relapsed or refractory diffuse large B-cell lymphoma. Biomark Res.

[36] Ilieva K, Eberl M, Jaehrling J, Blair D, Patra-Kneuer M, Boxhammer R, Alvarez Arias D (2023). Preclinical study of CD19 detection methods post tafasitamab treatment. Front Immunol.

[37] Fitzgerald KN, Quesada AE, von Keudell G, Raj S, Lewis NE, Dogan A, Salles G (2022). CD19 epitope masking by tafasitamab leads to delays in subsequent use of CD19 CAR T-cell therapy in two patients with aggressive mature B-cell lymphomas. Leuk Lymphoma.

[38] Merrill MH, Redd RA, Lambert N, Caimi PF, Pullarkat P, Godby RC, Bond DA (2025). Outcomes of tafasitamab and lenalidomide in large B-cell lymphoma based on prior CD19-directed CAR T exposure. Hemasphere.

